# High Resolution Genetic Mapping by Genome Sequencing Reveals Genome Duplication and Tetraploid Genetic Structure of the Diploid *Miscanthus sinensis*

**DOI:** 10.1371/journal.pone.0033821

**Published:** 2012-03-16

**Authors:** Xue-Feng Ma, Elaine Jensen, Nickolai Alexandrov, Maxim Troukhan, Liping Zhang, Sian Thomas-Jones, Kerrie Farrar, John Clifton-Brown, Iain Donnison, Timothy Swaller, Richard Flavell

**Affiliations:** 1 Ceres, Inc., Thousand Oaks, California, United States of America; 2 Institute of Biological, Environmental & Rural Sciences (IBERS), Aberystwyth University, Gogerddan, United Kingdom; University of Massachusetts Amherst, United States of America

## Abstract

We have created a high-resolution linkage map of *Miscanthus sinensis*, using genotyping-by-sequencing (GBS), identifying all 19 linkage groups for the first time. The result is technically significant since *Miscanthus* has a very large and highly heterozygous genome, but has no or limited genomics information to date. The composite linkage map containing markers from both parental linkage maps is composed of 3,745 SNP markers spanning 2,396 cM on 19 linkage groups with a 0.64 cM average resolution. Comparative genomics analyses of the *M. sinensis* composite linkage map to the genomes of sorghum, maize, rice, and *Brachypodium distachyon* indicate that sorghum has the closest syntenic relationship to *Miscanthus* compared to other species. The comparative results revealed that each pair of the 19 *M. sinensis* linkages aligned to one sorghum chromosome, except for LG8, which mapped to two sorghum chromosomes (4 and 7), presumably due to a chromosome fusion event after genome duplication. The data also revealed several other chromosome rearrangements relative to sorghum, including two telomere-centromere inversions of the sorghum syntenic chromosome 7 in LG8 of *M. sinensis* and two paracentric inversions of sorghum syntenic chromosome 4 in LG7 and LG8 of *M. sinensis*. The results clearly demonstrate, for the first time, that the diploid *M. sinensis* is tetraploid origin consisting of two sub-genomes. This complete and high resolution composite linkage map will not only serve as a useful resource for novel QTL discoveries, but also enable informed deployment of the wealth of existing genomics resources of other species to the improvement of *Miscanthus* as a high biomass energy crop. In addition, it has utility as a reference for genome sequence assembly for the forthcoming whole genome sequencing of the *Miscanthus* genus.

## Introduction

The ability to sequence whole genomes has advanced the means of detecting genetic variation genome wide. However, for large genomes that are exceptionally difficult to unambiguously assemble, genetic maps are still needed for genetic studies and plant breeding. Recently, several studies have used genotype-by-sequencing (GBS) to develop genetic maps, but usually on smaller and/or better understood genomes [1]–[4]. In this work we sought to create a high density genetic map of the large and unknown genome of *Miscanthus sinensis* using next generation sequencing (NGS) of a large number of heterozygous recombinants to open up the genetic mapping of the species and to support breeding studies.

*Miscanthus* is one of the leading biofuel crops with growing importance [5]. The genus *Miscanthus* Anderss. (Andropogoninae: Poaceae) consists of many rhizomatous perennial species, most of which are endemic to subtropical and tropical regions of southern Asia, with a few species extending to temperate eastern Asia [6]–[8]. This extensive distribution provides a wealth of genetic diversity and traits for *Miscanthus* improvement programs. Species showing the greatest potential as dedicated energy crops include *M. floridulus*, *M. lutarioriparium*, *M. sacchariflorus*, *M. sinensis* and *M.*×*giganteus*, among which, *M.*×*giganteus* is currently the most cultivated species for biomass production in Europe [9]–[10].

However, *M.*×*giganteus* is not an ideal candidate for genetic studies and breeding improvement due to its sterility and triploid genome (2n = 3*x* = 57) resulting from a rare natural cross between diploid *M. sinensis* (2n = 2*x* = 38) and allotetraploid *M. sacchariflorus* (2n = 4*x* = 76) [11]–[14]. Since one of the two genomes of the tetraploid *M. sacchariflorus* was inherited from the diploid *M. sinensis*, *M.*×*giganteus* has two genomes with high homology, and a third genome with low homology to *M. sinensis* [11], [12], [15]. Although the two parental species have populations showing substantial genetic diversity [6], [16], only a few different clones of *M.*×*giganteus* are available and have been widely cultivated through vegetative propagation [6], [17].

*M. sinensis* and *M. sacchariflorus*, on the other hand, have also been introduced and adapted widely to Europe as potential bioenergy species due to their high biomass potential [10]. These two species are therefore crucial to enhancing the genetic variability of *Miscanthus* crops and generating new high performance hybrids [18]. Of the two parental species, *M. sinensis* has the most widespread geographical distribution, in terms of latitude, longitude and altitude, with correspondingly high adaptability from extensive genetic and phenotypic diversity. It is also generally diploid in nature, demonstrating greater amenability to hybridization. Recent studies have demonstrated the existence of variation in flowering times in *M. sinensis* that provides extensive options for hybrid generation and subsequent optimization of varieties to different climatic zones [19].

Research for the majority of species within the *Miscanthus* genus is focused mainly on field trials, positioning knowledge of its basic biology behind that of current row crops. Little is known about the genetics behind important agronomic traits that must be improved for commercialization. Better understanding of the genetics of biomass yield related traits such as spring emergence date, flowering time control, senescence, nutrient uptake, and abiotic and biotic stress tolerances, is needed to aid genetic improvement of the crop. This in turn can be facilitated by a reference linkage map.

The small numbers of molecular studies on *Miscanthus* genomes to-date have concentrated on defining phylogenies and taxonomies using limited genetic marker technologies [6]–[8], [15], [17]. Researchers are still in the early stages of developing marker tools for *Miscanthus* for advanced genome studies [20]–[24]. The nuclear size of *M. sinensis* is about 2.75 pg/1C or 2,650 Mbp, which is approximately the same size as the maize (*Zea mays* L.) genome (2,500 Mbp) [14], [25]. The large and highly heterozygous genome of *Miscanthus* has caused considerable difficulties in developing genetic markers and linkage maps of the species. At present, there is only one published linkage map for *Miscanthus*, in *M. sinensis*, composed of 257 random amplified polymorphic DNA (RAPD) markers spread over 28 linkage fragments with half of the linkage fragments containing 2–4 markers only [26]. Although this singular genetic map has been used for quantitative trait loci (QTL) mapping of a number of combustion traits [27], [28], the discovered markers linked to the QTL are not easily used in marker-assisted breeding as the linkage map was built with non-sequence based RAPD markers. This lack of DNA sequence information prohibits comparative genomics analyses among other genome resources of well-studied crops, such as sorghum (*Sorghum bicolor* L.), maize and rice (*Oryza sativa* L.).

It is known that a complete *Miscanthus* linkage map covering all chromosomes is needed for a variety of genetic studies, such as QTL and whole genome association mapping. A complete map would also enable use of QTL accessible from other grass species through alignment based on syntenic relationships. In this work, we report the first complete, high resolution genetic map of *M. sinensis* and use of comparative genomics to understand its syntenic relationships with other species, particularly sorghum.

The linkage map was constructed based on several thousand single nucleotide polymorphism (SNP) markers using a mapping population that is composed of progeny of a “two-way pseudo-testcross”, full-sib family, which is typically used for genetic mapping of highly heterozygous out-crossing plant species [29]. The mapping progeny were genotyped using the Illumina next-generation sequencing platform [1], [2], [30], [31]. GBS is a robust approach for diverse species with large genomes [31], and has proven to be highly efficient for genotyping the mapping population of *M. sinensis* in the present work. A significant benefit of GBS is that genome-wide marker representation is ensured, which is critical for coverage and completeness of the linkage map. The linkage maps created from this study include two parental maps (female and male) and a composite map, created after confirming that the two parent maps had the same (or similar) structures detected by common markers. Once the *M. sinensis* linkage maps were aligned to the sorghum genome, two-to-one syntenic matches were observed between the 19 *M. sinensis* linkage groups and the 10 sorghum chromosomes, except for one fusion, proving a tetraploid nature of the diploid *M. sinensis*. Therefore, this linkage map not only defines all 19 chromosome linkage groups for the first time, but also provides a bridge for comparative genomics to genetic resources of other crops species for the ultimate improvement of *Miscanthus*.

## Results

### Genotyping by Sequencing

**Figure 1:**
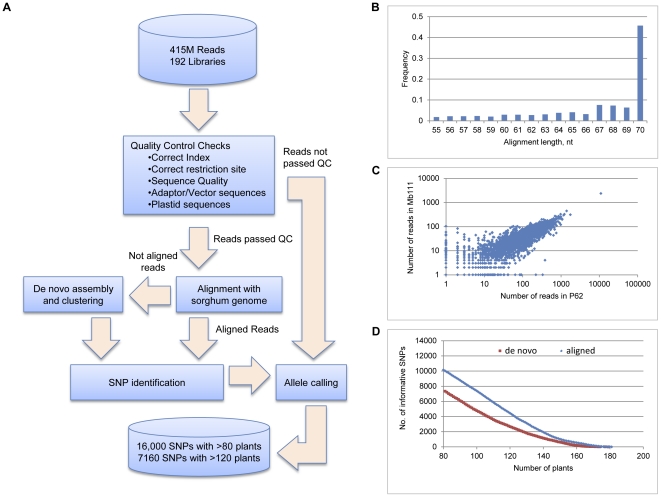
Sequence analysis and single nucleotide polymorphism (SNP) marker calling. (A) Processing workflow of the NGS data analysis. (B) Distribution of the alignment lengths for *Miscanthus* reads matching the sorghum genome. (C) Correlation between the numbers of reads per restriction site for two plants: Mb111 and P62, having the largest number of reads. Only reads mapped in chromosome 1 of sorghum were used as an example. Similar correlations were observed for almost all other pairs of plants and other chromosomes. (D) Coverage of SNPs by plants.

In order to focus SNP development on gene-rich space, a methylation sensitive restriction enzyme *Fse*I, which cleaves gene-rich regions of the genome, was used to construct reduced representation DNA libraries. After DNA sequencing using the Illumina platform, the main steps of processing sequencing data are presented in Figure 1A. The total number of sequencing reads produced was 415,694,046. Quality control (QC) was conducted on all reads by checking for the proper sequence layout consisting of a barcode sequence followed by a partial restriction site. In addition, the read was required to have quality scores greater than or equal to 15 on at least 80% of its nucleotides. The number of reads passing the QC steps was 106,291,299 (26% of the total number) with a majority of the reads filtered out due to the lack of proper layout (41% of reads passed this test) and sequencing quality (64% passed the test).

The number of reads varied in libraries from 328,450 to 6,470,626. All reads passing QC steps were mapped onto the sorghum genome using NCBI BLAST+. Only the best BLASTN match, having at least 90% identity and being at least 55 nucleotides long, was considered (almost half of matches covered the entire read, Figure 1B). A total of 23% of the reads passing QC matched the sorghum genome with these criteria.

The aligned reads from the most representative library of the female parent, Mb111, covered about 1,200,000 nucleotides in the sorghum genome. Approximately 788,000 nucleotides were covered by five or more reads, and from these, 23,753 nucleotides were polymorphic (SNP). The coverage of reads varied significantly for different restriction sites. Most of the restriction sites were covered by a very small number of reads, whereas a few restriction sites generated a very large number of reads. The number of reads per restriction site correlated between the libraries indicating that restriction sites had different accessibilities (or potentially different copy numbers) (Figure 1C).

We considered a nucleotide position to be homozygous in a plant if there was coverage of at least five reads in the corresponding library, having a nucleotide at this position with a quality score ≥15 and at least 90% of these nucleotides were the same. A position was considered to be heterozygous in a plant if there were at least five reads having a nucleotide at this position with a quality score ≥15 and there were two different alleles with frequencies between 0.4 and 0.6.

According to these two definitions, in an average plant, 97.8% of the sampled nucleotide positions with five or more reads were homozygous, 0.7% positions were heterozygous, and 1.5% positions were undetermined. We identified a total of 49,007 SNPs using this set of data.

Reads which did not match the sorghum genome were clustered using CLC Bio software into 20,169 contigs. For plant Mb111 as an example, this resulted in 1,432,240 additional positions with coverage of at least five reads. An additional 13,897 SNPs were identified from this dataset using the same criteria as the reads mapped to sorghum. To get better coverage of the discovered SNPs by as many plants as possible, we used sequences previously excluded at the QC step to increase the number of reads in defined SNP positions. We considered all discovered SNPs with at least four reads and defined the plant as homozygous if the frequency of a certain nucleotide in this SNP was ≥0.9; heterozygous if there were two different nucleotide variants with frequencies between 0.2 and 0.8. Special codes were then assigned to those SNPs having nucleotide frequencies between 0.8 and 0.9.

In further analyses we considered only SNPs with major allele frequency in the population less than 0.8. As a result, we obtained 4,437 SNPs aligned and 2,669 SNPs not aligned to the sorghum genome, making a total 7,106 SNPs, with determined alleles in at least 120 plants (Figure 1D). Eleven plants were excluded for further mapping analyses as they contained unknown alleles of most SNPs. In both aligned and *de novo* SNPs the most frequent allele variations were complementary pairs of mutations A/G and C/T, which occurred about twice more than the next allele pair, C/G.

### Marker segregation analyses

The SNP markers were then coded for JoinMap and 5,600 out of the 7,106 SNPs were selected for segregation and mapping analyses. More heterozygous markers were identified in the female than in the male. Of the 5,600 markers, 2,574 (46%) were heterozygous only in the female; 1,468 (26%) only in the male; and 1,558 (28%) were heterozygous in both parents. Chi-square tests indicated that 54% (1,394 out of 2,574) of the female markers and 49% (715 out of 1,468) of the male markers showed segregation distortion (goodness-of-fit ratio 1∶1, α = 0.05), as did 69% (1,078 out of 1,558) of the female and male common markers (goodness-of-fit ratio 1∶2∶1, α = 0.05). Thus, markers that were heterozygous in both parents tended to be more distorted in allele transmission than when heterozygous in only one of the parents.

### Linkage mapping

The coded data were first used to create parental maps using two JoinMap populations, one using markers segregating in the female, and the other in the male [29], [32], [33]. The markers that were heterozygous in both parents were used in both parental maps and served as common markers to match homologous linkages between the two. As expected, 19 linkage groups were identified for each parent.

For linkage mapping, framework maps of both parents were created first with no (p<0.05) segregation distorted markers, and used for homologous identification and structural comparisons between the two parental maps. One-to-one homologous relation was confirmed using markers common between the 19 linkage groups of the female and the 19 linkage groups of the male. In addition, good collinearities were observed and no obvious structural heterogeneities were seen between homologous framework maps of the two parents.

Then, segregation distorted markers were added to the framework maps by attempting different LOD statistics and/or different numbers of markers. The final maps selected were usually the ones having the best collinearities with the framework maps. Since the female had more heterozygous markers than the male, the number of markers mapped in each female linkage was generally more than that in the corresponding linkage of the male (Table 1).

**Table 1:** Mapping data summary of the parental and the composite maps.

Miscanthus LG	Sorghum Syntenic Chr	Female map			Male map			Compose map			Mapped to syntenic sorghum chr		Mapped to other sorghum chr		Not mapped to sorghum chr	
		No.	Length (cM)	Avg (cM)	No.	Length (cM)	Avg (cM)	No.	Length (cM)	Avg (cM)	No.	%	No.	%	No.	%
1	1	196	139.6	0.71	108	111.1	1.03	226	132.9	0.57	140	61.9	14	6.2	72	31.9
2	1	208	160.6	0.77	132	161.7	1.22	276	150.8	0.66	187	67.8	12	4.3	77	27.9
3	2	201	147.0	0.73	214	144.5	0.68	261	136.8	0.52	167	64.0	5	1.9	89	34.1
4	2	237	140.2	0.59	171	138.1	0.81	268	136.8	0.59	159	59.3	33	12.3	76	28.4
5	3	197	145.9	0.74	76	140.0	1.84	220	153.1	0.73	133	60.5	18	8.2	69	31.4
6	3	180	143.3	0.80	104	146.7	1.41	193	152.4	0.79	127	65.8	4	2.1	62	32.1
7	4	166	127.7	0.77	134	140.0	1.04	219	130.0	0.57	139	63.5	5	2.3	75	34.2
8	4, 7	233	165.2	0.71	232	164.1	0.71	289	158.8	0.55	184	63.7	14	4.8	91	31.5
9	5	134	124.2	0.93	59	94.9	1.61	149	129.0	0.87	40	26.8	23	15.4	86	57.7
10	5	110	113.1	1.03	42	122.8	2.92	131	107.5	0.82	39	29.8	7	5.3	85	64.9
11	6	182	117.5	0.65	77	105.0	1.36	219	115.8	0.53	155	70.8	13	5.9	51	23.3
12	6	135	101.7	0.75	109	96.6	0.89	176	104.6	0.54	132	75.0	7	4.0	37	21.0
13	7	126	115.7	0.92	85	125.0	1.47	143	123.1	0.86	88	61.5	19	13.3	36	25.2
14	8	103	101.4	0.98	100	98.2	0.98	172	113.4	0.65	72	41.9	14	8.1	86	50.0
15	8	109	113.1	1.04	77	106.0	1.38	139	111.2	0.80	89	64.0	12	8.6	38	27.3
16	9	118	111.8	0.95	63	126.9	2.01	150	112.8	0.75	63	42.0	37	24.7	50	33.3
17	9	130	94.8	0.73	95	80.3	0.85	190	91.1	0.47	108	56.8	9	4.7	73	38.4
18	10	137	127.7	0.93	66	121.8	1.84	167	133.5	0.80	111	66.5	7	4.2	49	29.3
19	10	139	100.3	0.72	64	94.0	1.47	157	102.2	0.84	80	51.0	17	10.8	60	38.2
Total		3041	2390.7	0.79	2008	2317.4	1.15	3745	2395.6	0.64	2213	59.1	270	7.2	1262	33.7

Since there were no obvious structural heterogeneities between the female and male maps, a composite map containing both female and male markers was created to facilitate future QTL mapping of the population. The composite mapping was done with an independent JoinMap mapping population by simultaneous analysis of all polymorphic markers from both parents. Again, 19 linkage groups were formed and framework maps without segregation distorted markers were created first. Comparisons between the composite and the two parental framework linkages showed perfectly collinear marker order agreements for all 19 linkage groups, indicating good integration of the two parental linkages by the simultaneous mapping analysis.

**Figure 2:**
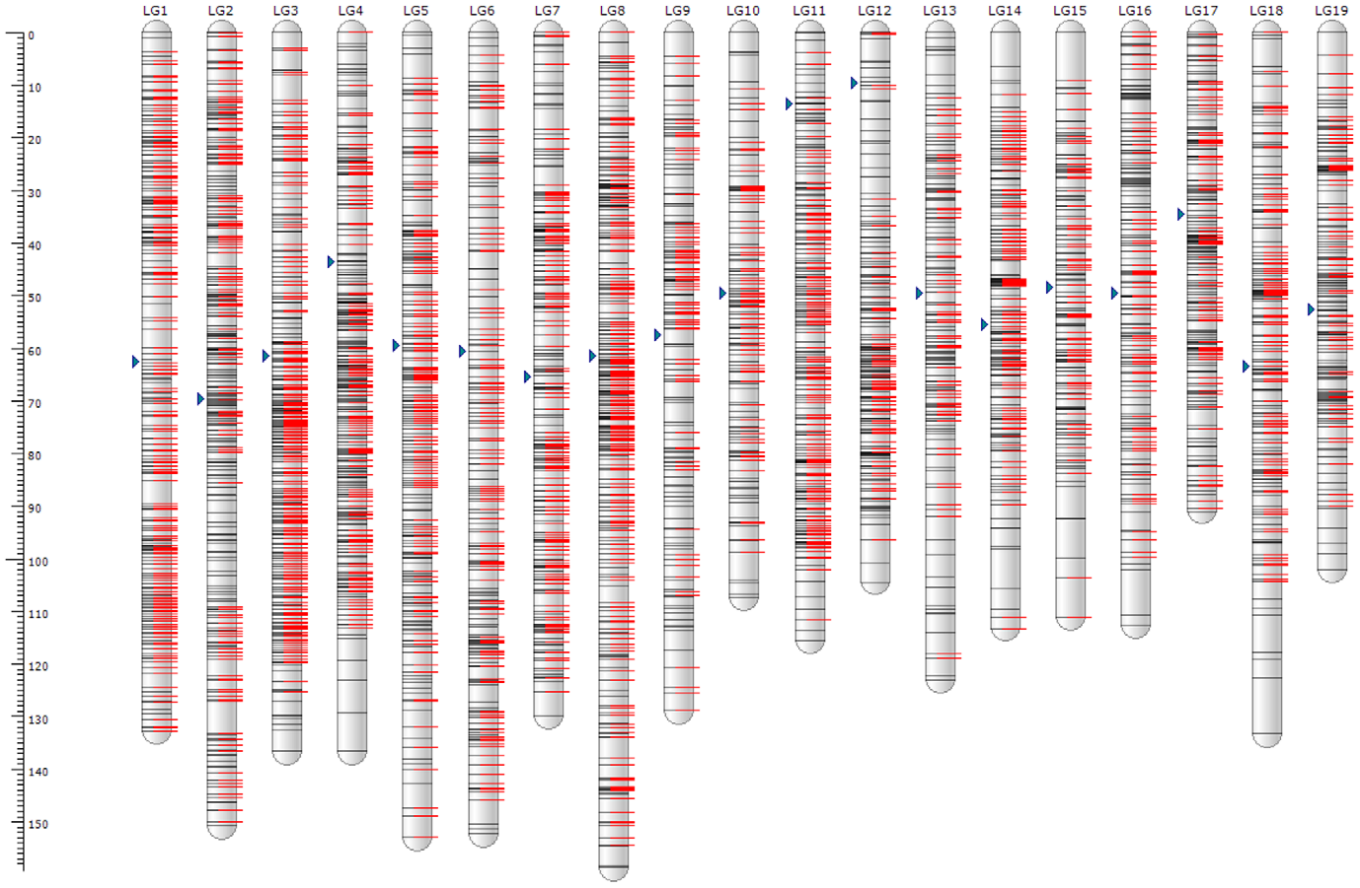
Linkage length and marker distribution of the composite genetic map. The linkage groups (LGs) are named from LG1 to LG19 in agreement with their syntenic relationship to the already defined 10 sorghum chromosomes. On each linkage, the gray or black lines represent mapped markers; the right-shifted red lines signify framework markers. The triangle next to each linkage group represents tentative centromere position of the linkage. The details of the composite map were given in Table S1.

The composite framework maps were then extended by mapping segregation distorted markers using the same mapping criteria and strategy as the parental maps. Ultimately, a high density genetic linkage map representing all chromosomes was established (Table 1, Table S1 and Figure 2). The linkages were named LG1 to LG19, following the syntenic relation order of sorghum chromosomes, described in a separate section below.

The composite map comprised 3,745 SNP markers spanning 2396 cM on 19 linkages. The numbers of markers mapped varied among the 19 linkage groups from 131 markers in LG10 to 289 in LG8. The linkage sizes in cM were also very different, from 91 cM of LG17 to 159 of LG8. The marker density of the composite map was highly variable from region to region and chromosome to chromosome, with an average resolution of 0.64 cM (Table 1, Table S1 and Figure 2).

### Map validation

**Figure 3:**
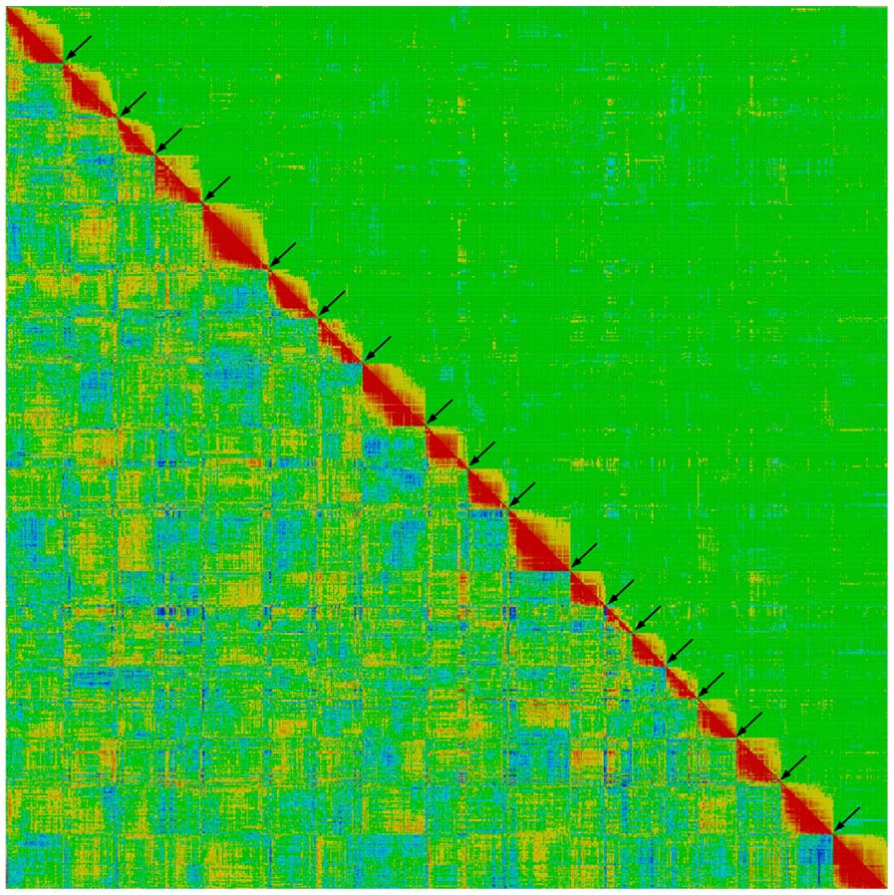
Graphical representation of the high quality linkage map of *M. sinensis*. The image is produced with CheckMatrix (http://www.atgc.org/XLinkage/) to validate and verify the quality of the composite map using BIT score (low-left diagonal) and REC score (top-right diagonal). Red color represents tight linkage; yellow represents weak linkage; green to blue represents no linkage. The red along the diagonal, but lack of red off the diagonal, indicate that marker assignments and orders in the 19 linkage groups are supported by both JoinMap and CheckMatrix.

GBS has been used in plants recently, but to the best of our knowledge, this is the first time this technology has been applied in genetic linkage mapping involving a large number of plants for such a large unknown genome species, without any reference sequence of the species itself. Thus, sequence and SNP calling accuracy and mapping quality needed to be carefully addressed. For this study, an independent program, CheckMatrix (http://www.atgc.org/XLinkage/), was used to validate the linkage map (Figure 3). All mapped markers, including segregation distorted markers, were included for CheckMatrix validation. A red color in CheckMatrix represents tight linkage, thus for each linkage a red diagonal should be observed if all markers were in the correct order. Any markers that were found unfit in their positions were remapped using JoinMap with different LOD statistics or removed from the final maps until all marker positions were accepted by both JoinMap and CheckMatrix. After each linkage was verified, a CheckMatrix of all 19 linkage groups was plotted using 25% of the mapped markers of each linkage (Figure 3). Overall results indicated that all markers mapped in the composite map were assigned to correct linkages and placed in the correct order.

### Comparative genomics

**Figure 4:**
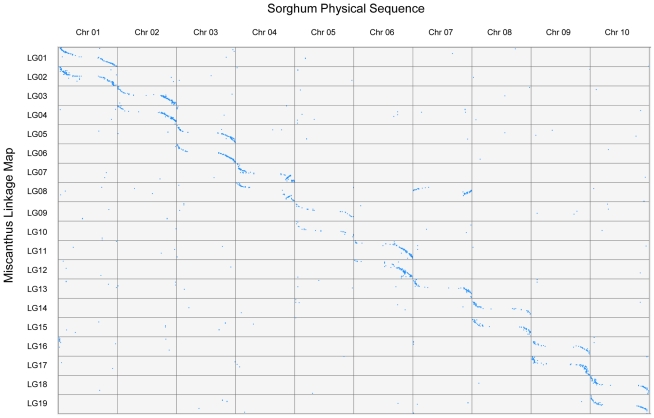
Syntenic alignment between the 19 linkage groups of *M. sinensis* and 10 chromosomes of sorghum. The big gap in each chromosome plot corresponds to centromere heterochromatic region that inhibits recombination. The image shows genome-wide sequence duplications and chromosome rearrangements in *M. sinensis* compared to sorghum.

**Figure 5:**
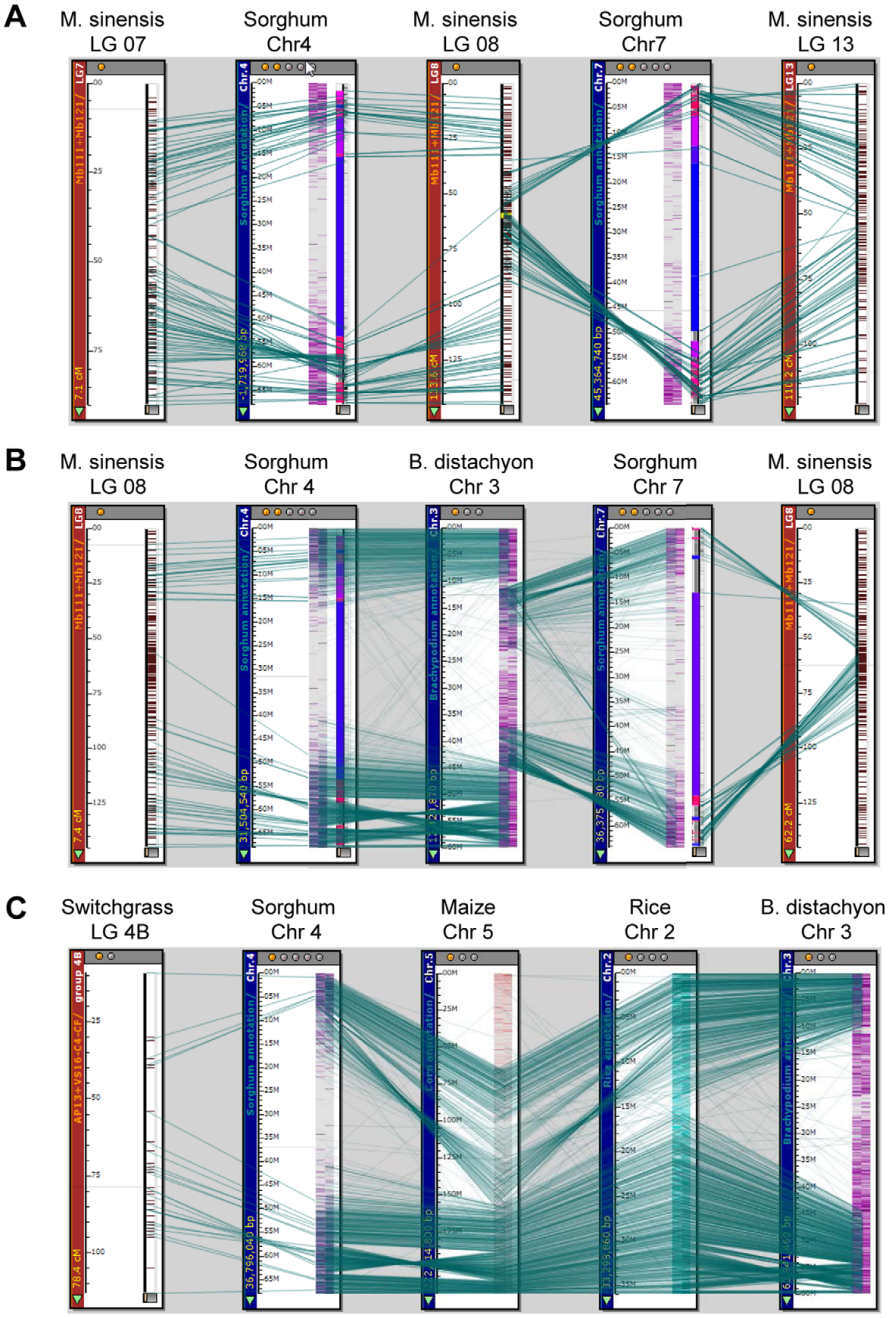
Chromosome structural rearrangements of *M. sinensis* when compared to sorghum and several other species. (A) Chromosome fusion in LG8 of *M. sinensis* was observed between two sorghum syntenic chromosomes 4 and 7; chromosome 7 is inserted in the middle of chromosome 4 in telemore-centromere inverted order. However, the other duplicated copies of chromosomes 4 and 7 are not fused, corresponding to LG7 and LG13 in *M. sinensis*, respectively. Also, a common paracentric inversion in the long arm of the syntenic chromosome 4 was seen in both the fused copy (LG8) and the non-fused copy (LG7) of *M. sinensis*. (B) The fusion of sorghum syntenic chromosomes 4 and 7 in *M. sinensis* LG8 was also similarly observed in chromosome 3 of *B. distachyon*. (C) The same paracentric inversion between the long arm of the sorghum syntenic chromosome 4 and *M. sinensis* LG7 and LG8 was also seen when sorghum was compared to switchgrass, rice, and maize, but only partially seen when compared to *B. distachyon*.

The marker sequences used in this study were aligned to the genome sequences of sorghum, maize, rice, and *B. distachyon* using BLAST+. Most sequence conservation existed between *M. sinensis* and sorghum (Figure 4). The sequence alignments of the *M. sinensis* linkage map to the three other species (maize, rice, and *B. distachyon*) were poor because the ratios of BLAST alignments were very low in comparison to the sorghum alignment. Moreover, there was a two-to-one syntenic match between the 19 *Miscanthus* linkages and the 10 sorghum chromosomes, indicating complete, genome-wide chromosome duplication, with one exception: one of the syntenic copies of sorghum chromosomes 4 and 7 was fused into a single linkage group, LG8, in *Miscanthus*, thus making 19 basic chromosomes of the *Miscanthus* genus (Figure 4). The syntenic copy of the sorghum chromosome 7 fragment of LG8 was inserted in the middle of syntenic chromosome 4, with a telomere-centromere inverted order, while two segments corresponding to the syntenic chromosome 4 at the two ends of LG8 were still collinear with sorghum chromosome 4, but with another paracentric inversion in the long arm of the syntenic chromosome 4 (Figure 4 and Figure 5A). The syntenic sorghum chromosome 4 and 7 fusion in LG8 of *M. sinensis* was also similarly observed in *B. distachyon* (Figure 5B).

The same paracentric inversion as in LG8 was also seen in LG7, which is the other syntenic copy of sorghum chromosome 4, although this copy did not fuse with chromosome 7 (Figure 4 and Figure 5A). Further comparative analyses involving sorghum, maize, rice, *B. distachyon* and switchgrass indicated that the paracentric inversion between the sorghum chromosome 4 and the *M. sinensis* syntenic linkages (LG7 and LG8) occurred in sorghum, not in *M. sinensis*, because the same paracentric inversion was also observed when sorghum was compared to switchgrass, maize and rice, and also was partially seen when compared to *B. distachyon* (Figure 5C). Comparative analysis also indicated that every linkage of *M. sinensis* has markers unmapped to sorghum, or mapped to non-syntenic chromosomes of sorghum (Table 1 and Figure 4) as a result of evolutionary changes, including transpositions.

It is evident that the diploid *M. sinensis* has a tetraploid genome structure that consists of two sub-genomes, each syntenic to the entire sorghum genome, with a few major structural rearrangements of the chromosomes. Therefore, the *M. sinensis* linkages were named from LG1 to LG19 according to the synteny and chromosome order of the 10 sorghum chromosomes (Table 1 and Figure 4).

## Discussion

*M. sinensis* has a large genome that is close to the size of the maize genome and, as we discovered, also has genome-wide duplication. Repeats in genomes complicate interpretation of the segregation of genetic markers and make the search for informative SNPs particularly challenging. GBS permits simultaneous genotyping and marker discovery, allowing large-scale genotyping to be done without prior marker development [1], [2], [30], [31].

Using a subset of the discovered SNP markers, a high-density composite linkage map covering all 19 linkage groups was constructed for the first time for *M. sinensis*, and also for the *Miscanthus* genus. Accuracy of marker placements in the linkage groups has been carefully tested in multiple ways in the present work.

First, linkage analyses were done for each parent separately. Two parental maps, each containing 19 linkage groups, were created in advance and inspected for discrepancies. Comparison of the parental framework maps showed collinearity between common markers of all 19 linkage groups, suggesting identical chromosomal structure in both parents.

Second, the composite map was built independently by “simultaneous analysis” of markers that were used in the parental maps, rather than by merging the two maps by “map integration” process. An advantage of simultaneous analysis is that markers can be selected using segregation information, producing an initial framework map free of any segregation distorted makers. In addition, linkage maps obtained by simultaneous analysis can be visually inspected through graphical genotyping of the mapping codes in JoinMap.

Third, the composite map was inspected by comparison to the framework map and the two parental maps. In addition, due to uncertainties resulting from missing values or potential errors in SNP calling for reads from duplicated DNA sequences, markers were removed from mapping if they gave ambiguous positions between different mapping analyses. The use of CheckMatrix to inspect marker ordering through graphical genotyping provided effective feedback to validate the linkage analyses of JoinMap. Overall results suggested correct linkage grouping and ordering of the markers in the composite map.

The linkage length and number of markers of the *M. sinensis* genetic map varied greatly among different linkages. The eight linkages (LG1 to LG8) that were syntenic to sorghum chromosomes 1, 2, 3 and 4 generally contained more markers and also longer linkages (in cM) than other linkage groups (Table 1). A similar marker distribution was observed in the switchgrass genetic map where the first eight linkage groups (1A, 1B to 4A and 4B), syntenic to sorghum chromosomes 1–4, were also larger than other chromosome linkages [34]. The *M. sinensis* and switchgrass genetic maps also share other common features. For example, the two *M. sinensis* linkages (LG9 and LG10) syntenic to sorghum chromosome 5 contain the least markers and the lowest percentages (<30%) of markers that map to sorghum, however markers which mapped to other linkages exhibit much higher percentages (60% on average) of sorghum syntenic alignments (Table 1), again, similar to when the switchgrass genetic map was aligned to sorghum. The data also suggest that linkages syntenic to sorghum chromosome 5 have encountered more evolutionary changes than other chromosomes after the species diverged from each other. A similar result was recently documented in maize, of which the two ancestral maize chromosomes orthologous to sorghum chromosome 5 retain the smallest number of syntenic orthologs to sorghum genes [35].

The marker sequences were mapped to genome sequences of several grass species. Among grasses with sequenced genomes (rice, sorghum, maize, and *B. distachyon*), sorghum has the closest phylogenetic relationship to *Miscanthus*. As predicted, the *Miscanthus* DNA reads better matched the sorghum genome than the maize genome, which in turn was a better match than the rice and *B. distachyon* genomes. Similar findings were reported recently using a survey sequence of *M.*×*giganteus* [22].

In the current study, a detailed relationship was established for the first time between *Miscanthus* and sorghum. Our map indicates that the current diploid *M. sinensis* evolved from genome duplication of its progenitor that was very close to a sorghum ancestor, bearing 10 haploid chromosomes. As a result, the current *M. sinensis* still maintains two genome subsets, each containing a complete syntenic set of its progenitor genome or half of the current diploid *M. sinensis* genome. A similar genome structure has been seen in maize, which is also a segmental tetraploid originating from a tetraploidization event that was reported to have occurred several million years ago [35], [36].

Marker sequence alignments to the sorghum genome suggest end-to-end complete chromosome coverage of all 10 sorghum chromosomes (Figure 4). As expected, very few markers from the *M. sinensis* linkages were aligned to sorghum centromeric regions due to low frequencies of *Fse*I cleavage and SNP polymorphism around the centromeric heterochromatic regions. The overall results suggest that the composite map has a genome-wide coverage of gene-rich, euchromatic regions of *M. sinensis*. High marker coverage of the genome in this study enabled robust linkage mapping analysis and a comprehensive genome structure study using syntenic relationships to other grasses.

The current composite map will also serve as an important bridge to allow *Miscanthus* researchers to use genomic resources of sorghum, as well as many other well studied species that already have syntenic relationship established to sorghum, thus greatly extending the genomics tools that can be used for *Miscanthus* improvement. In addition, the present map should provide a good reference for *Miscanthus* sequence assembly due to its high resolution and quality. The map will also be used for QTL discoveries of traits of importance for biomass production in *Miscanthus*.

## Materials and Methods

### Mapping population

An outcrossing full-sib F1 mapping population (JoinMap CP population type) consisting of 230 genotypes was produced between two parental lines, female Mb111 (synonyms EMI11 and MS-88-110) and male Mb121 (synonym H0023). Parent Mb121 is one of the progenies derived from a cross between siblings (F1.1 and F1.7) that originated from another cross between MS-90-2 and Mb111 [26]–[28]. Therefore, the male parent Mb121 is a second generation progeny of the female parent Mb111. Seeds were germinated in JI3 compost (John Innes Manufacturers Association, Harrogate, UK) in an unlit glasshouse in January and February of 2006. Seedlings were then transferred into 10″ pots for 12 months before the plant rhizome was split to produce viable clones. In May 2007, one clone from each progeny was planted into each of three replicated randomized blocks, at a site near Aberystwyth (52°26′N, 3°59′W), on the west coast of Wales. This mapping population, named Mx2, segregates for a number of important traits, including flowering time, plant height, stem number, senescence, spring emergence, and biomass yield. Mx2 is thus an ideal population for QTL studies of biomass related traits in *M. sinensis*.

### DNA isolation and library construction

A total of 192 (out of the 230) genotypes of the mapping population, including parents, were selected for genotyping using the GBS platform [1], [2], [30], [31]. Genomic DNA (gDNA) was isolated from dried leaf tissue using the DNeasy 96 Plant Kit (Qiagen). Then, 500 ng DNA of each sample was digested with *Fse*I enzyme (New England Biolabs (NEB®), Hitchin, UK) at 37°C for 3 h; subsequently the enzyme was heat-inactivated at 65°C for 20 min.

Twelve unique P1 adapters were designed, each with a different 6 bp barcode to enable a 12× sample multiplex per Illumina flow cell lane. Each *Fse*I digested DNA sample was ligated to one of the P1 adapters in a 20 µl reaction mixture containing 1 µl of 10 nM adapter, 1 µl rATP (100 mM, Promega), 1 µl NEB buffer 4, and 0.5 µl T4 ligase (400,000 cohesive end units/mL, NEB) by incubating for 4 h at 16°C. The reaction was stopped by incubating at 65°C for 20 min. Every 12 samples, each with a unique barcode, were pooled and randomly sheared by Bioruptor UCD-300 (Diagenode) to an average size of 450 bp. The barcoded samples were electrophoresed on 1.5% agarose gels, and fragments of 300–500 bp were recovered and purified. The fragments were treated with T4 DNA polymerase, T4 polynucleotide kinase, and Klenow DNA polymerase (NEB) for end repairing, followed by treatment with Klenow exō (NEB) and dATP (Invitrogen) to generate 3′ adenine (A) overhangs [1], [2], [30], [31].

A common P2 adapter containing thymine (T) overhangs (Illumina) was ligated to sheared, size-selected, P1-ligated and pooled DNA templates with 1 µl of 100 nM adapter and T4 ligase for 4 h at 16°C [1], [2], [30], [31]. The samples were purified and eluted to 50 µl. PCR enrichment of the pooled libraries were performed in 50-µl PCR reactions (30 s at 98°C, followed by 15 cycles of 10 s at 98°C, 30 s at 65°C, 30 s at 72°C, and a final extension step of 5 min at 72°C). PCR amplicons were electrophoresed on 1.5% agarose gels, and fragments of 300–500 bp were recovered and purified. The pooled libraries were loaded onto a 2100 Bioanlyzer (Agilent) for quality control, evaluating fragment size and appearance of adapter dimers. The libraries were sequenced using an Illumina GAIIx instrument following standard protocols.

### Sequence analysis and SNP marker calling

The DNA sequences were produced using two Illumina flow cells, consisting of eight lanes per cell and a multiplex of 12 samples per lane. DNA sequence reads were 76 nucleotides, with the first six nucleotides barcode sequence specific for given samples within the lane and the next six nucleotides partial restriction sites for *Fse*I. DNA sequences were analyzed using the NCBI BLAST+ program for mapping reads into the sorghum genome sequence and CLC BIO software for *de novo* assembly of reads having no match with sorghum. An internally developed proprietary Oracle-based program was used for quality control, marker discovery, allele calling and data storage. All *Miscanthus* DNA sequences from this study were submitted to the National Center for Biotechnology Information (NCBI) Short Read Archive (study SRA050103).

### Mapping code assignment

The SNP markers were coded following the coding scheme of CP population type of JoinMap 4 [33]. Three kinds of segregation types were involved, including markers that were heterozygous only in the female (lmxll), only in the male (nnxnp), or in both parents (hkxhk). Although the majority of data were co-dominantly coded, dominant codes were also applied for uncertain genotyping values of hkxhk type.

### Marker selection

To ensure linkage map quality, the SNP markers used for final mapping were selected by removing markers that (A) showed differences of allele frequencies between the first 96 plants and the second 96 plants due to a potential library preparation bias between the two 96-well gDNA plates or a sequencing quality bias between the two flow cells; (B) had more than 60 missing values if they were heterozygous in only one of the parents (segregation type lmxll or nnxnp), or more than 30 missing values if they were heterozygous in both parents (hkxhk); (C) showed segregation distortion with *x* ^2^>100 of goodness-of-fit tests; and (D) contained identical recombinant information. Eventually 5,600 SNPs were selected and imported into JoinMap for linkage mapping analyses.

### Linkage mapping analyses

Marker data were analyzed with JoinMap 4 using the CP population type [33]. To ensure that any potential chromosome structure variations between the two parents were captured, maternal and paternal maps were first analyzed following the two-way pseudo-testcross strategy [29]. After observing that there was no obvious structural heterogeneity between the female and male chromosome linkage groups, a composite map was generated by simultaneous analyses of all markers from both parents. Therefore, three independent linkage analyses including female, male and composite mapping, were performed. The two parental maps assisted in validating the composite map.

Markers were assigned to correct linkage groups using two-point grouping analyses in three steps. First, only markers that showed un-distorted segregation (goodness-of-fit ratio 1∶1 if segregating in only one parent, or 1∶2∶1 if segregating in both parents, α = 0.05) were assigned into linkage groups at the minimum independence test LOD score of 12. Second, ungrouped, including segregation distorted, markers were assigned to existing linkage groups based on “strongest cross link (SCL)” with a minimum LOD threshold of 10. Third, markers from different groups that showed strongest cross link were combined until no strongest cross link was seen between markers from different groups. After these three steps, the expected 19 linkage groups were formed for all three linkage analyses including the female, the male, and the composite mapping.

Markers within each group were mapped using the regression mapping algorithm with the minimum LOD score of 1.0 to 5.0 and maximum recombination frequency of 0.35 or 0.40 depending on the linkage group [32], [33]. Map distance was estimated using the Kosambi mapping function. For each linkage group, a framework map containing no segregation-distorted markers was created first; segregation-distorted markers were added to the linkage using the framework map as a reference. In addition, at least five independent mapping analyses of each group were performed to compare if marker orders were relatively consistent between different LOD statistics. Any markers that showed dramatic discrepancy of their relative position between different LOD statistics were excluded from further mapping. The final map selected exhibited the most agreement in marker order with the framework map.

### Mapping validation

Linkage maps were all visually inspected by graphic genotyping in the JoinMap program. Due to potential sequencing errors and uncertainties caused by missing values and sequence duplication, the qualities of the final maps were carefully addressed and validated by an independent program CheckMatrix using PyMap BIT and REC scores as described by Kozik (http://www.atgc.org/XLinkage/). Any markers that were detected as having weak linkage positions were remapped by adjusting JoinMap parameters, or removed from final mapping.

### Comparative genomics

Using NCBI BLAST+ the DNA sequences containing the SNPs of interest were aligned to the genomes of sorghum, rice, maize and *Brachypodium distachyon* (L.). Only BLASTN matches longer than or equal to 55 nucleotides with identity greater than or equal to 90% were used. The alignment to sorghum was performed using raw sequence reads of *M. sinensis* as described in the sequence analysis and SNP calling section; however, the alignment to other species used only sequences that produced SNP markers. The syntenic genome relationships of *M. sinensis* to other species were visualized using an internally developed visualization and comparative genomics software Persephone (Ceres, Inc.). In addition, a dot matrix plot was generated with Persephone using linkage map positions of *M. sinensis* markers against aligned sequence positions of the sorghum genome, allowing a whole genome comparison view of the two species.

## Supporting Information

Table S1 **Composite map and genotype data of the mapping population.**
(XLS)
